# WHO framework on meaningful engagement: A transformational approach to integrate lived experience in the noncommunicable disease and mental health agenda

**DOI:** 10.1371/journal.pgph.0002312

**Published:** 2024-05-29

**Authors:** Jack Fisher, Guy Fones, Yvonne Arivalagan, Ida Ahmadpour, Svetlana Akselrod, Maia Olsen

**Affiliations:** 1 World Health Organization, Global Coordination Mechanism for the Prevention and Control of Noncommunicable Diseases, Geneva, Switzerland; 2 Center for Global Health, Department of Neurology, School of Medicine and Health, Technical University of Munich, Munich, Germany; 3 World Health Organization, Global NCD Platform, Geneva, Switzerland; Qatar University College of Medicine, QATAR

## Abstract

As the global public health community continues to reflect and recover from the COVID-19 pandemic, noncommunicable diseases (NCDs), and mental health and neurological conditions remains one of the largest unmet gaps in progress towards meeting the Sustainable Development Goals (SDG). People living with these health conditions, particularly among those most marginalized, acutely understand the impact of these failures in global action and investment. Integrating lived experience into the NCD and mental health response can act as an accelerator for action. Through a participatory process to co-create the WHO Framework on Meaningful Engagement on NCDs, and Mental Health and Neurological Conditions, we conducted a mixed methods and semi-structured approach, including informal consultations, focus groups, in-depth interviews, online surveys, and a short film series, that captured the perspectives of 700 individuals from 111 countries, including 386 individuals with lived experience. Working alongside lived experience communities and other relevant stakeholders, we have established and co-created a set of principles, enablers and actions for operationalizing meaningful engagement, related to dignity and respect, power and equity, inclusivity and intersectionality, commitment and transparency, and institutionalization and contextualization. People with lived experience have a right to be equitably included in all levels of policy-setting, design and implementation of programs, and to have a central role in reforming and reorienting the structures and systems intended to address the complex multifactorial challenges that they face. WHO is committed to leveraging its role in global health to further operationalize meaningful engagement within WHO and its Member States.

## Introduction

Noncommunicable diseases (NCDs), and mental health and neurological conditions constitute one of the most significant, and most neglected, global disease burdens. As of 2019, the World Health Organization recorded that 17 million people died from an NCD before they reached 70 years of age, which accounted for 74% of all global deaths [1]. One in eight individuals was living with a mental health condition [2]. Neurological conditions are the leading cause of disability-adjusted life years (DALYs) and the second leading cause of death globally, accounting for nine million deaths per year [3]. Global trends over the past three decades, driven by demographic changes and population ageing, with population prevalence of NCDs, mental health and neurological conditions expected to increase [4].

Low- and middle-income countries (LMICs) experience a high share of the global burden, accounting for 77% of all NCD deaths, including 86% of premature deaths under the age of 70 [1]. Similarly, 77% of all suicides [5] and 78·5% of neurological disorders worldwide [3] were also in LMICs. Further, LMICs also struggle with workforce capacity and access to affordable, quality care–where for instance, the workforce for neurological health is 70 times smaller than in high-income countries [6]. The COVID-19 pandemic has further exacerbated these health inequities as well as strained already weak and fractured health systems, where people living with chronic health conditions have been disproportionately impacted by disruptions in health care services due to the pandemic, particularly among low-income populations and high-risk and vulnerable communities [7]. Of 130 countries investigated in a recent WHO rapid assessment on service delivery for NCDs during the COVID-19 pandemic, 93% of countries reported health service disruptions due to COVID-19 during the third quarter of 2022 [8, 9].

Most countries are off track on the Sustainable Development Goal (SDG) targets to reduce by one-third premature mortality from NCDs and promote mental health and well-being (SDG target 3·4) and to ensure responsive, inclusive, and participatory decision-making at all levels (SDG target 16·7) [10]. People living with NCDs, and mental health and neurological conditions often experience unfair and harmful exclusion and marginalization due to a variety of intersectional factors and social constructs, including socioeconomic status, ethnicity, gender identity, sexual orientation, disability, age, nationality, or immigration status [11]. It has been widely demonstrated that oppression and discrimination result in negative health outcomes, including preventable health conditions and exclusion from accessing affordable, life-saving treatment and care. Social inequities also impact who sets agendas and who participates in the policy-setting process, and power asymmetries and structural drivers must be addressed to accelerate progress for the furthest left behind and to promote health equity [12].

Individuals with lived experience of NCDs, and mental health and neurological conditions understand challenges inherent to the related burden of these health challenges intimately and have developed an expertise through their lived experience. As such, individuals with lived experience should be meaningfully engaged to co-create and implement policies, programs and services that directly impact their lives. This can also lead to more equitable, sustainable, and person-centred solutions which can improve health outcomes, leading to stronger health systems and a greater return on health investments.

The WHO Global Coordination Mechanism on the Prevention and Control of Noncommunicable Diseases conducted a mixed-methods and participatory approach including consultations, focus groups, key informant interviews, online surveys, and a documentary film series with people with lived experience across all WHO geographic regions. This led to the better understanding of lived experience communities, how individuals viewed the concept of meaningful engagement, the role of WHO and Member States as strategic stakeholders, resulting in co-creating a set of principles and practices for operationalizing meaningful engagement. All elements of the two-year program and study design were co-created and informed by individuals with lived experience, alongside representatives from WHO, Member States and non-state actors. The results and recommendations of this work were recently published as the WHO Framework for Meaningful Engagement of People Living with NCDs, and Mental Health and Neurological Conditions [13] (hereby referred to as the Framework).

Meaningful engagement is defined by the World Health Organization as “respectful, dignified, and equitable inclusion of individuals with lived experience in a range of processes and activities within an enabling environment where power is transferred to people, valuing lived experience as a form of expertise and applying it to improve health outcomes [13].” Meaningful engagement can further empower and enable individuals with lived experience to participate in the “enjoyment of the highest attainable standard of health” as “one of the fundamental rights of every human being without distinction of race, religion, political belief, or economic or social condition”, as defined in the Constitution of the World Health Organization [14].

Various examples from other areas of health informed the process to develop the Framework. “Continuum” models such as Sherry Arnstein’s “Ladder of Citizen Participation” (which has since been amended by various scholars and activists, including Rosalind Eyben and David Wilcox) informed the structure of the consultation and co-creation process [15–17] and helped set expectations that these efforts in NCDs, mental health and neurological conditions intended to go well beyond the lowest tiers of limited participation and tokenism. HIV and disability rights groups have effectively leveraged rights-based approaches in the past, led by communities with lived experience to pursue legal protection against discrimination and stigmatization, push for more inclusive and equitable access to health care, dismantle systems of power and address social inequities such as education, employment, and housing [18, 19]. They have also influenced policy change at the highest political level, led and inspired by a call to action of those living with HIV and AIDs in declarations like the 1983 Denver Principles [20].

In this article, we explore how the perspectives of people with lived experience can provide a blueprint for meaningful engagement in co-creating solutions that can address the global burden of NCDs, and mental health, and neurological conditions.

## Methods

The perspectives of 700 individuals (hereby referred to as Participants) from 111 countries were captured through various methods from September 2020 to October 2022, including informal consultations, focus groups, in-depth interviews, online surveys, and a documentary film series. A narrative review was also conducted, which revealed the lack of prior evidence on the impact for meaningful engagement of individuals with lived experience in NCDs, and mental health and neurological conditions and a need for increased formal knowledge and evidence of impact in this field. All individuals who participated in individual activities provided consent through written consent forms and/or completed WHO due diligence processes and procedures for engaging with WHO as individual external experts. Participatory research approaches related to the development of the WHO Framework were reviewed and approved by Technical Expert Networks (TENS) comprised of senior leadership at WHO headquarters, WHO regional offices, and WHO country offices, but was not initially designed as an academic research study subject to IRB approval.

### Participation, inclusion, and iterative methodology

We used a semi-structured and iterative approach, incorporating participation and input from individuals with lived experience throughout all elements of data collection. Our guiding methodology focused on four iterative and overlapping phases: 1) listening and learning from the lived experience community, 2) collecting initial data and ideating additional steps in the study process, 3) analyzing and reviewing qualitative data and community input, and 4) consolidating findings and strategizing recommendations for the Framework. Cultivating an inclusive approach to participation was also a central tenet to the study design. Participants were recruited through open calls for participation in online advertisements, social media, WHO newsletters and websites, and through snowballing sampling methods working with collaborating organizations to identify potential participants, in order to capture as many perspectives as possible in the regional and online consultations. For methods such as key informant interviews where a smaller set of participants were pre-selected and screened, we focused on maintaining a diverse and inclusive balance of perspectives across regions, countries, conditions, gender, ethnicity, age, and other socioeconomic and demographic factors.

As comprehensive and intentional as efforts were to develop the Framework across regions and practitioners, there were some communities and experiences that were inevitably missing from this analysis. Consultations skewed towards lived experience experts who were already integrated into global and regional networks, spoke one of the six UN languages, had access to internet and bandwidth that allowed virtual participation, had time to set aside for consultations, and whose health status allowed them to volunteer their time. More commitment is needed to ensure that the perspectives of the communities most marginalized are considered in future efforts and that meaningful engagement is inclusive to all. This includes continual efforts to actively ensure representation and meaningful engagement with groups such as those living in poverty, in rural or under-resourced communities, Indigenous and First Nation populations, ethnic and minority groups, people living with disabilities, and people of different ages, such as adolescents, children and older adults.

### Informal consultations with people living with NCDs

From December 2020 to May 2022, nine informal consultations or forums on meaningful engagement were conducted across six WHO regions and included 272 individuals with lived experience alongside representatives from WHO, Member States and other relevant non-state actors [21]. The first informal consultation in December 2020 was global in focus, which included 185 participants and a total of 55 people with lived experience and provided a template for further consultations at global and regional level. From February to May 2022, nine informal regional consultations and forums were held across all WHO regions; Table 1 provides additional information on participation in these consultations.

**Table 1:** Representation of participants and member states from the six informal regional consultations with people living with NCDs, mental health and neurological conditions from February to May 2022.

	African Region (AFRO)		Region of the Americas (PAHO)—*North America and Caribbean*		Region of the Americas (PAHO)—*Central and Latin America*		European Region (EURO)		Eastern Mediterrean Region (EMRO)		South-East Asian Region (SEARO)*	
**Total participants**	274		105		116		82		165		135	
**Total participants who identified as representing the region**	217	79,2%	82	78,1%	41	35,3%	36	43,9%	116	70,3%	95	70,4%
**Total participants who identified as having lived experience in NCDs**	46	16,8%	16	15,2%	25	21,6%	25	30,5%	21	12,7%	28	20,7%
**Total participants who identified as having lived experience in mental health conditions**	40	14,6%	10	9,5%	9	7,8%	13	15,9%	8	4,8%	6	4,4%
**Total participants who identified as representing an indigenous group**	24	11,1%	3	3,7%	6	14,6%	4	11,1%	11	9,5%	9	9,5%
**Member States represented—low and middle-income countries**	27 (Benin, Botswana, Burkina Faso, Cameroon, Republic of the Congo, Côte d’Ivoire, Eswatini, Ethiopia, Gambia, Ghana, Guinea, Kenya, Liberia, Mali, Mauritius, Mozambique, Niger, Nigeria, Rwanda, Sénégal, South Africa, Sudan, Tanzania, Togo, Uganda, Zambia, Zimbabwe)		7 (Belize, Dominica, Grenada, Haiti, Jamaica, St. Lucia, St. Vincent and Grenadines)		14 (Argentina, Bolivia, Brazil, Colombia, Costa Rica, Cuba, Dominican Republic, Ecuador, El Salvador, Guatemala, México, Nicaragua, Paraguay, Perú)		9 (Armenia, Belarus, Bosnia and Herzegovina, Georgia, Kazakhstan, Kyrgyzstan, Russian Federation, Tajikistan, Ukraine)		12 (Algeria, Egypt, Iran, Iraq, Jordan, Lebanon, Libya, Morocco, Pakistan, Palestine, Tunisia, Yemen)		9 (Bangladesh, Bhutan, India, Indonesia, *Malaysia*, Myanmar, Nepal, Sri Lanka, Thailand)	
**Member States represented—high-income countries**	1 (Seychelles)		11 (Aruba, Antigua and Barbuda, The Bahamas, Barbados, Canada, Curaçao, Guyana, St. Kitts and Nevis, St. Martin, Trinidad and Tobago, United States)		2 (Chile, Panama)		16 (Belgium, Cyprus, France, Germany, Greece, Ireland, Lithuania, Luxembourg, Poland, Portugal, Romania, Spain, Sweden, Switzerland, The Netherlands, United Kingdom)		5 (Bahrain, Oman, Qatar, Saudi Arabia, United Arab Emirates)		1 (*Hong Kong SAR*, *China*)	

Participant data regarding lived experience of NCD and mental health conditions was self-identified through an online registration process and may be underreported and/or double counted across the two categories. Representation with an indigenous group was also self-identified data collected at registration. Income groups for low- and middle-income countries and high-income countries are based on 2023 World Bank classifications [21]. Authors indicate an

* in the participant data collected from the SEARO consultations, since participants from three countries in the Western Pacific (WPRO) region were included in consultation records. The three informal regional forums held by WHO in the Western Pacific (WPRO) region in Cambodia, Malaysia, and the Philippines referenced elsewhere in this paper are not included in Table 1, given significant differentiation in organization of the informal forums and collection of participant data; see Table 2 for more information.

**Table 2:** Participant information, timeline, recruitment, selection, co-design principles, and types of data collected for all participatory approaches utilized in the development of the WHO framework for meaningful engagement throughout December 2020 to November 2022.

	Timeline	Number of sessions	Total participants	Total participants identified with lived experience	Regions represented	Member States represented	Languages represented	Format	Recruitment	Selection of participants	Participatory and co-design elements	Type of data collected	Contributions to WHO Framework
**WHO Global Informal Consultation with People Living With NCDs**	December 2020	1	185	55	All	n/a	English	Two half-day sessions	Invitations to WHO, UN, WHO Civil Society Working Group, and relevant civil society organizations	Selection based on completion of a Declaration of Interest form, per WHO’s Framework of Engagement with Non-State Actors	• Co-chaired by people living with NCDs • Most speakers had lived experience • Interactive breakout sessions • “Pass-the-mic” opportunities for facilitated group discussion	• Polling of participants during the consultation • Qualitative data from interactive breakout session discussions	• Provided template for informal regional consultations in 2022 • Qualitative insights published in WHO *"Nothing For Us*, *Without Us"* report (April 2021) • Identified themes for further research and activities, including development of WHO Framework for Meaningful Engagement
**Focus Group discussions on meaningful engagement**	November 2021	6	35	35	AFRO, EMRO, EURO, PAHO, WPRO, SEARO	18	English	One 90 minute session per 10–12 participants	Screening and invitations from WHO based on lived experience and past participation	Selection based on completion of the Declaration of Interest process	Opportunities for participants to indicate focus group topics of most interest	Qualitative data collected from transcripts from interviews, conducted through semi-structured guided questions	• Qualitative insights published in WHO *Intention to Action* series ("*People Power"*, April 2023) • Identified themes and principles highlighted in WHO Framework for Meaningful Engagement
**In-Depth Interviews on meaningful engagement**		n/a	12		AFRO, EMRO, EURO, PAHO, WPRO, SEARO	n/a	English	One 90 minute session per two interviewees, 6 in total	Invitations from WHO for further exploration of themes to select individuals involved in focus groups	Selection based on completion of the Declaration of Interest process	Facilitated opportunities for interactive discussion led by The Social Kinetic	Qualitative data collected from transcripts from interviews, conducted through semi-structured guided questions	• Qualitative insights published in WHO *Intention to Action* series ("*People Power"*, April 2023) • Identified themes and principles highlighted in WHO Framework for Meaningful Engagement
**WHO Informal Regional Consultations on Meaningful Engagement**	February—May 2022	6	437	247	AFRO, EMRO, EURO, PAHO, SEARO	114	English (all), Spanish (PAHO)	Three half-day sessions	Self nominations to the consultation through expression of interest form and outreach through WHO and civil society networks, invitations to informal consultative groups to co-design workshops through nominations by the WHO regional office based on lived experience and past participation	Selection based on completion of the Declaration of Interest process	• Co-designed and co-hosted by people living with NCDs • Majority of speakers had lived experience • Interactive breakout sessions • “Pass-the-mic” opportunities for facilitated group discussion	• Polling of participants during the consultation • Qualitative data from interactive breakout session discussions	• Analysis from informal consultations published in WHO *Intention to Action* series ("*Regional Reflections"*, April 2023) • Identified themes and principles highlighted in WHO Framework for Meaningful Engagement
**WHO Western Pacific Regional Forums with People Living with NCDs**	May 2022	3	25	25	WPRO	3	English, Khmer, Tagalog	One 3 hour session	Invitations from WHO Country Offices in each country to organized local or national groups of people with lived experience	Selection based on completion of the Declaration of Interest process	Opportunities for participants to indicate focus group topics of most interest	• Quantitative data from multiple choice or Likert scale survey responses • Qualitative data from open-ended survey questions	• Analysis from informal consultations published in WHO *Intention to Action* series ("*Regional Reflections"*, April 2023) • Identified themes and principles highlighted in WHO Framework for Meaningful Engagement
**Online survey on enhancing meaningful engagement with people living with NCDs**	July—August 2022	n/a	280	280	All	n/a	English	Online survey	Request for participation sent from WHO to individuals who had previously participated in activities related to the WHO Framework	All received responses were reviewed by WHO	Open-ended questions to solicit feedback on activities related to the development of the WHO Framework	• Quantitative data from multiple choice or Likert scale survey responses • Qualitative data from open-ended survey questions	Informed next steps for WHO in developing and implementing the WHO Framework and related meaningful engagement activities
***"Nothing For Us*, *Without Us"* Documentary Film**	September 2022	n/a	65 submitted responses	6	All	6	Arabic, English, Portuguese, Nepali	2 days of pre-production and filming	Invitations from WHO to a select group of individuals based on past participation, or nomination by WHO Country or Regional Offices	Selection of interviewees based on lived experience and representation across WHO regions and across conditions and marginalized communities	Individuals being filmed helped develop and shape their personal narratives through the filming and interview process	Qualitative insights through filmed interviews	Interviews were published in the WHO documentary film *"Nothing For Us*, *Without Us"* film released in September 2022
**Web consultation on the zero draft of the WHO Framework on Meaningful Engagement**	October-November 2022	n/a	65 submitted responses	18	AFRO, EMRO, EURO, PAHO, WPRO, SEARO	3	English	Online submission	Open request online for written feedback to the zero draft of the WHO Framework	All responses and comments received by WHO were reviewed and considered in revisions to WHO Framework	• Open call for feedback used to revise final version of the WHO Framework • Open invitation webinar hosted by WHO to share received feedback and invite further feedback and discussion	Written comments and suggested in-text edits to the zero draft of the WHO Framework	Community comments and responses were considered in revisions to WHO Framework prior to final publication of the document

Contributions of each approach to the development and co-design of the Framework and additional publications is summarized in final column and is further discussed in Results and Discussion.

All global and regional consultations and forums were multistakeholder in nature, participatory and co-facilitated by lived experience advocates–both independent contributors and those linked to civil society organizations, ensuring a safe and inclusive space. Consultation sessions included presentations, breakout groups, and “pass-the-mic” sessions and focused on the value, principles, and preferred definitions of meaningful engagement, which informed the WHO’s definition of meaningful engagement in the finalized Framework. Consultations also focused on strategies, tools, and practices for operationalizing meaningful engagement. Qualitative insights from the consultations were collected through in-session surveys and through recordings and note-taking analyzed after each event, and qualitative content informed the themes, principles, and enablers featured throughout the WHO Framework. A WHO publication in the WHO *Intention to Action* series was also released in April 2023 on regional reflections and priorities in activating meaningful engagement [22].

### Focus groups, in-depth interviews, and other evidence generation activities

In November 2021, WHO held a series of semi-structured focus group discussions with 35 individuals from 18 countries. Participants were screened and invited to participate based on previous participation in informal consultations, with attention to diversity of perspectives across geographic regions and representation across populations and demographic factors, including gender, age, related health condition and lived experience(s). Focus group discussions included 6–8 participants and were 90 minutes in duration, using a semi-structured interview guide developed and facilitated by the consultancy firm, The Social Kinetic. From the larger group of focus group participants, 12 individuals with lived experience were invited for a further set of in-depth interviews. Each of the six in-depth interviews were 90 minutes in duration with two individuals, with each of the interview pairs focusing on one of six key themes that were identified in the focus group discussions, which included: 1) advocacy and human rights, 2) community engagement across broader health networks and health systems, 3) exclusion and the importance of involving groups that are marginalized, 4) informed decision-making and health literacy, 5) lived experience as evidence and expertise, and 6) power dynamics and power reorientation towards individuals with lived experience [23]. A similar set of key informant interviews were conducted with seven WHO staff, and 12 specialists across international and non-governmental organizations. Qualitative insights from the focus group discussions were published separately in a WHO *Intention to Action* series publication in April 2023 [23]; with both the qualitative insights from the in-depth interviews and focus groups included throughout the WHO Framework.

A documentary film was released in September 2022, featuring interviews with six individuals with lived experience in diabetes, rheumatic heart disease, mental health conditions, cancer, and disabilities. Participants were pre-selected by WHO to represent a diverse cross-section of geographic regions and lived experience. Throughout the Framework development process, one-off online surveys were also utilized to further garner community feedback. The WHO Secretariat used an iterative sampling and analysis approach, whereby the lessons learnt from each activity were used to review, adjust and test the approaches for subsequent activities. The findings and data that emerged from each activity were analysed by both using deductive and semantic approaches, grouped into thematic areas and consolidated to inform evidence-based positions for the framework. From October to November 2022, a web consultation on the zero draft of the Framework was conducted, which was an open invitation for lived experience participants and other stakeholders to provide written feedback over email to an early draft of the Framework over a three-week period. 65 responses were submitted from individuals representing WHO, governments, civil society, and individuals with lived experience, which included over 750 comments or suggested in-text edits for review.

## Results

**Fig 1:**
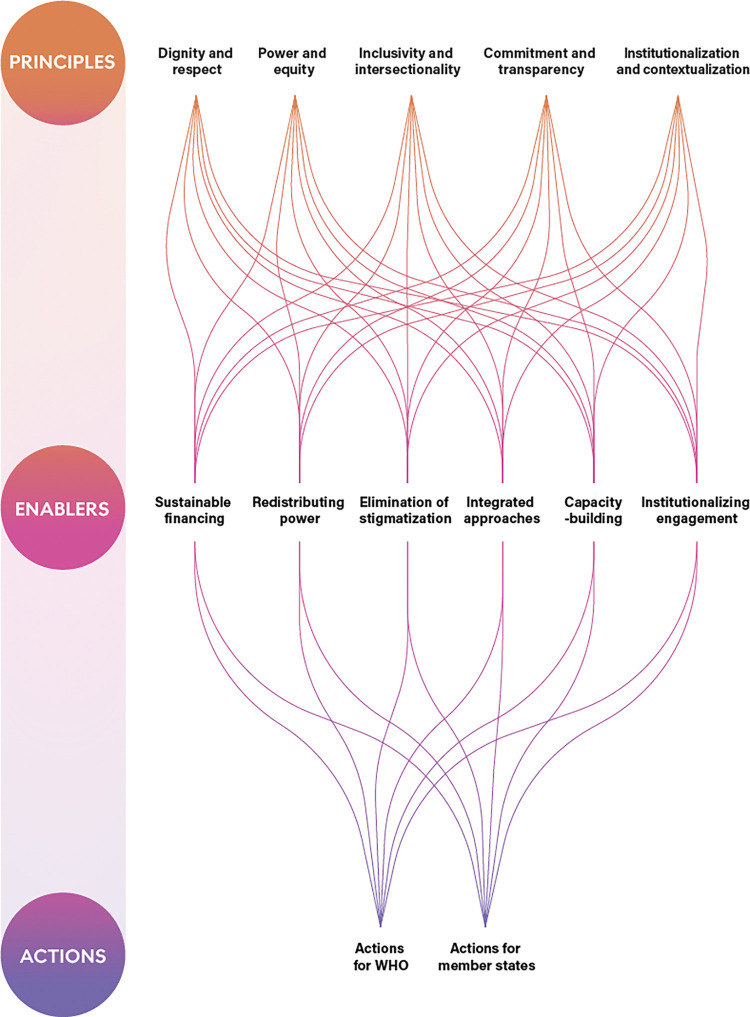
Interconnections between principles and enablers of meaningful engagement of people living with NCDs, and mental health and neurological conditions in policy-setting and programmes.

Through the participatory research that has been conducted in this initiative among people with lived experience of NCDs, and mental health and neurological conditions, a set of five principles and six enablers for operationalizing meaningful engagement emerged (Fig 1), which have been incorporated in the Framework. In addition to the set of 11 principles and enablers, an extensive set of actions have been recommended for WHO and Member States to incorporate in planning, implementation, and monitoring of future policies, programs, and services intended to address NCDs, and mental health and neurological conditions. These recommendations have been presented in a phased approach with three tiers of operationalization (bronze, silver, and gold), which allow measurable benchmarks for stakeholders to progressively scale up lived experience efforts over time.

### Principles and enablers of meaningful engagement

As referenced in Fig 1, the five principles of meaningful engagement that have been identified in the WHO Framework through the consultative process with individuals with lived experience are: 1) dignity and respect, 2) power and equity, 3) inclusivity and intersectionality, 4) commitment and transparency, and 5) institutionalization and contextualization. In addition to the five overarching principles identified in the Framework, a set of six enablers that can facilitate meaningful engagement were identified as 1) sustainable financing, 2) redistributing power, 3) elimination of stigmatization, 4) integrated approaches, 5) capacity building, and 6) institutionalizing engagement.

In consultations and activities related to the development of the Framework, participants noted the need for an approach that acknowledges **dignity and respect** of all individuals living with these health conditions and the right to attain the highest standard of health, without being stigmatized and treated as “patients” or people who are solely responsible for their health condition(s). Participants called for more inclusive, “person-first” language that highlights the *person* living with an NCD, mental health condition, or neurological condition rather than essentializing someone as their medical ailment or classification. Participants also consistently raised that they had experienced being blamed for their health condition, being overmedicalized or dehumanized in interactions in their community, or being discriminated against in accessing safe and appropriate health care, housing, and employment. As a result, the Framework recommends reviewing and reforming norms and practices within WHO and Member States and establishing clear, well-implemented legal frameworks to **reduce and mitigate stigmatization** and discrimination and to respect and protect the dignity and human rights of individuals living with NCDs, and mental health and neurological conditions.

Participants noted the pain and harm that they or others in their community have experienced through exclusion, discrimination, and stigmatization across systems and structures, including governance, policymaking, health care settings, schools, employment, and other institutional or community settings. Acknowledging **power and equity** and the ways in which discrimination, oppression, and health inequities can affect people with lived experience, and the importance of creating safe, **inclusive, and intersectional spaces** available for anyone with lived experience to contribute to meaningful engagement were central themes that emerged throughout consultations and interviews. Various recommendations in the Framework were thus established around how to equitably address systemic and structural power imbalances that have been barriers to engagement for people with lived experience and to open more opportunities for participation, ensuring a diversity of voices so that sexism, racism, heterosexism, ableism, and other forms of discrimination are not perpetuated. This may also mean “bringing the table” to specific communities, which is particularly pertinent in ensuring there is equitable inclusion of populations that are marginalized who may need additional support and access to participate fully. Commitments to **redistributing power** and practicing critical allyship through anti-racism, anti-oppression, anti-colonialism, and anti-discrimination lenses was also highlighted as key areas in the Framework, and closely informed activities throughout these efforts [24].

Participants also voiced frustrations over the economic costs, time, and personal vulnerabilities that are often undervalued or expected on a voluntary basis in order to contribute their lived experience, which is inequitable and disrespectful of their expertise. Lived experience approaches are often disproportionately under-resourced within programs and policy-setting efforts. As a result, the Framework sets a strong recommendation that meaningful engagement should be **sustainably financed** and that institutions should value the contributions of people with lived experience, equivalent to other areas of technical expertise. The Framework also outlines how meaningful engagement can be further ensured through **transparency** and consistency in communication, mitigation of conflicts of interest, and a **commitment** to clearly establishing goals and expectations around how contributions made by people with lived experience will be incorporated into governance, policy-setting, or programming.

Throughout consultations and activities, participants focused often on what made meaningful engagement *meaningful*, as opposed to inconsistent, hollow, or tokenistic approaches, and that institutions must be committed to ensuring that engagement efforts are doing more than “ticking off boxes” around inclusion of people with lived experience. Participants made the analogy that they expect not just to be “invited to the table” in a one-off fashion, but to have a substantial contribution at all levels of policy-setting processes and programming. Throughout consultations and interviews, participants regularly highlighted the need for meaningful engagement to be **institutionalized and integrated at all levels of policy and programming**, with opportunities for co-creation at all steps in the process, including governance, strategy, program design, implementation, and evaluation. As a result, the Framework sets one of the clearest calls to action for WHO and governments–“meaningful engagement must be formally integrated and embedded into all relevant programme areas and process to ensure sustained action and impact” [13].

Finally, participants noted that a critical step towards facilitating inclusive and diverse engagement across communities with lived experience is through **contextualizing solutions** to local settings and through **capacity-building,** training and knowledge exchange platforms. Participants often centered discussions on improving health literacy and knowledge of self-management as a foundation to a more empowered community. Beyond health literacy, the Framework also proposes investments in evidence-based capacity-building and skills development in advocacy, policy, rights-based approaches, communications, research, and other technical contributions that can better prepare lived experience advocates for meaningful engagement activities. Training and knowledge sharing opportunities should also be inclusive and accessible, particularly in underrepresented communities.

### Phased and regional approaches

**Fig 2:**
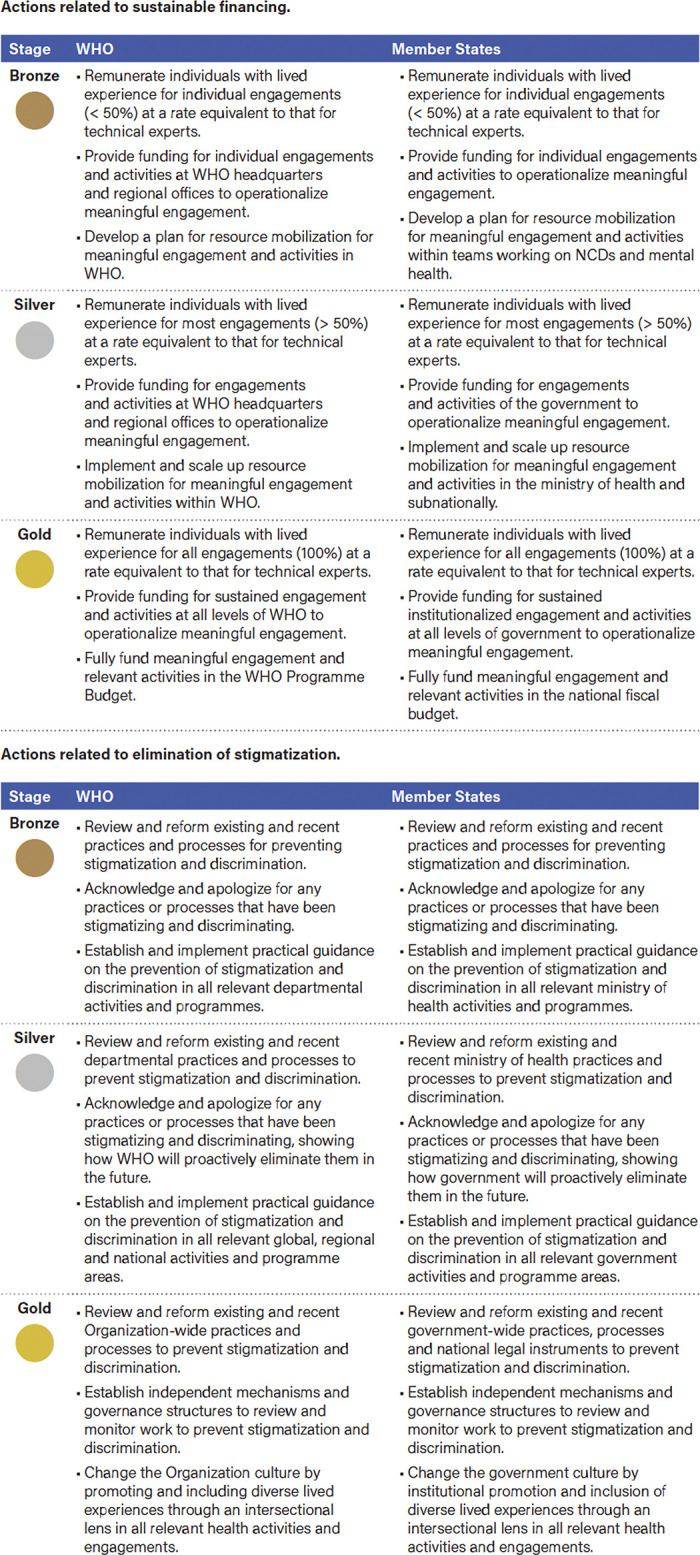
Examples of recommended actions from the framework.

Throughout the Framework, recommendations to WHO and Member States to operationalize meaningful engagement are provided for each enabler in a phased, stepwise approach with gold, silver, and bronze level benchmarks. An example of a phased approach to recommendations can be seen in Fig 2, and a full set of recommended actions can be found in the Framework. A phased approach is also intended to engage and respond to WHO Member States requests to maker progress towards the recommended actions of the Framework according to priorities and needs within regional contexts.

**Fig 3:**
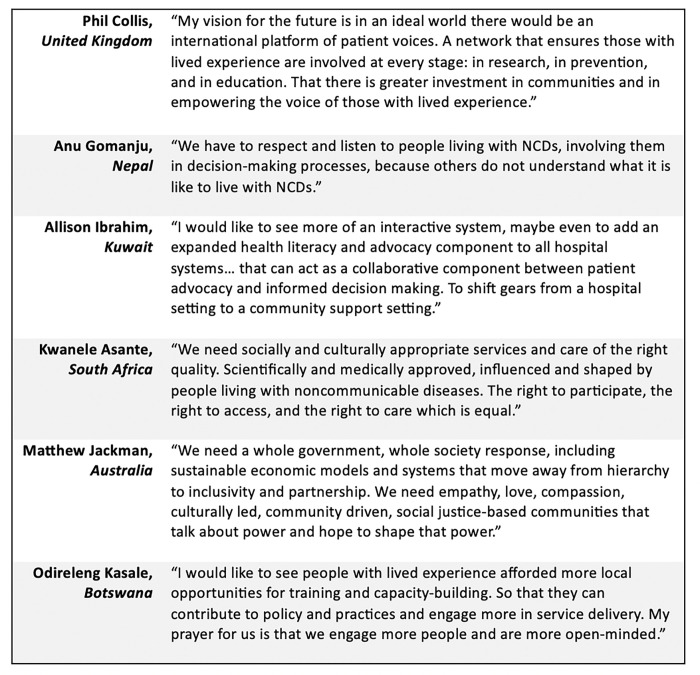
Lived experience quotes from participants.

There was broad consensus globally on the overlapping themes raised by participants across all regions that then mapped onto the 11 principles and enablers developed for the Framework. Yet, by conducting informal consultations and forums specific to each WHO region, participants were able to identify challenges and opportunities unique to their regional and cultural context. For example, while a commitment to greater inclusion of marginalized groups was a central topic in all consultations and activities, the populations that were identified as particularly vulnerable varied by region. The experience of refugees and migrants affected by NCD, mental health, and neurological conditions were at the forefront of discussions in eastern Mediterranean and Africa regional consultations but were not as consistently noted across other regions. Children were prioritized as a priority population for Latin America and the Caribbean and North America while older adults were the focus in Southeast Asia discussions. Opportunities for further action across regions also aligned around consistent global principles like reducing stigma and increased political commitments, but each region elevated a few unique priority areas, such as addressing burnout, establishing rights-based approaches among health care providers, prioritizing efforts around mental health, or developing contextual solutions in meaningful engagement that do not rely on the ‘Global North’ [22] (Fig 3).

## Discussion

The Framework was developed to provide practical guidance, norms, and standards for WHO and Member States to transition from intention to action in operationalizing meaningful engagement. The Framework is a significant signal of intent within WHO and its Member States on this topic. As the Framework demonstrates, an inclusive and intersectional approach to meaningful engagement can be achieved through various mechanisms, enablers, and recommendations, from diverse and transparent inclusion of underrepresented and marginalized groups, efforts to address stigmatization and inequitable power structures, providing sustainable financing and community-based support to facilitate sustained engagement, integrating the principles of meaningful engagement at every level of institutional operations and culture, and adaptation and contextualization to local settings so that meaningful engagement is accessible across languages, cultural norms, socioeconomic backgrounds, and political contexts.

Meaningful engagement and other participatory approaches should be seen not only as tools but as a rights-based approach to addressing health inequities and achieving health for all. The HIV/AIDS movement was influential in our understanding of participatory approaches and the potential benefit of integrating and institutionalizing meaningful engagement in global health. However transformative commitments are needed to include lived experience and reshape a more equity-driven and inclusive approach to policy-setting and implementation in NCDs, mental health and neurological conditions. To support this goal, the Framework provides practicable, achievable steps, and an adaptable and accessible approach to implementation across regions. In acknowledging the operational model of WHO, with regional and country offices leading implementation at the national level, the Framework allows flexibility to contextually evaluate, scale, and track commitments to meaningful engagement over time. All recommended actions aligning to the six enablers for meaningful engagement are presented with clearly defined and measurable bronze, silver, and gold benchmarks which allows governments to adapt a stepwise approach so that meaningful engagement can be strengthened and scaled over time. Another strength throughout the co-creation process was the opportunity to identify promising projects and collaborations, which are being led by organizations and lived experience advocates across regions. Evidence generation activities were able to bring together implementers in this space for the first time for knowledge exchange and discussion at a global and regional levels, and the Framework represents the depth of perspectives, experiences, and lessons learned across a diverse and inclusive set of stakeholders.

The Framework calls for new participatory models to be established or existing ones updated to promote a more fluid, balanced, and pragmatic approach to participation. This also acknowledges the power balance WHO holds within this context, ensuring more systematic and integrative approaches on this topic. The Framework is also aligned with WHO’s “triple billion targets” for achieving universal health coverage (UHC), addressing health emergencies, and promoting healthier populations by supporting the co-creation of contextually appropriate policies, programs, and services for NCDs and mental health globally [25, 26]. By integrating an intersectional lens, the Framework also has strong links to primary health care, healthy ageing, social and commercial determinants of health, gender and health, climate change, and humanitarian crisis.

Although meaningful engagement efforts have been demonstrated to be effective in improving health outcomes such as reducing hospital readmissions and improving adherence to treatment among young people and adults in mental health settings [27–31] or in longer survival, better clinical outcomes, higher quality of life, and a reduction of stigmatization for individuals living with HIV and AIDS [32–34] more evidence of impact is needed. The global health community has much to do in increasing the evidence base for the clinical and social benefits of meaningful engagement in NCDs, and mental health and neurological conditions. Future research should focus on operationalization, feasibility, and effectiveness of meaningful engagement and the impact that communities with lived experience can have on strengthening health systems.

Further, whilst meaningful engagement efforts can be inherently rights-based and equity-focused in their goals, even the most well-intentioned efforts can run the risk of leaving people behind. People with lived experience in NCDs, and mental health and neurological conditions represent a wide variety of perspectives across complex and intersecting determinants of health. There are various intersectional barriers that prevent equitable participation or representation in meaningful engagement, such as language, education, socioeconomic background, health or digital literacy, internet access, proximity to urban centers, exclusion due to gender, age, or ethnicity, limited knowledge of effective advocacy, and fatigue or burnout. In consultations and activities led by the WHO to develop the Framework, opportunities to participate were limited in by recruitment and self-selection bias to individuals with lived experience who were already well connected to WHO or established civil society networks, and/or who were comfortable and well versed in UN languages and in responding and contributing to complex outputs.

Efforts to improve health outcomes for people living with NCDs, and mental health and neurological conditions must also be integrated beyond the health sector, with action and commitment needed to improve education and employment opportunities, eliminate poverty, improve housing and living conditions, and end discrimination in all forms [35]. Relatedly, meaningful engagement cannot be siloed across health agendas and opportunities for alignment and shared learning can be found across many other areas of health and development. The Framework recommends that lived experience efforts related to NCDs, and mental health and neurological conditions are integrated across other communities and sectors, including HIV and AIDs, Tuberculosis, maternal and child health, UHC, healthy ageing, and disability.

WHO has made various commitments to continue forward the momentum around the recommended actions of the Framework. A series of “promising practices” case studies are being analyzed and published by WHO in 2024, which will provide further guidance on how to embed individuals with lived experience effectively into governance, programs, policy, and services. A conscious choice was made to term these projects “promising practices” rather than “best practices” to move away from the “one size fits all” set of operational guidelines for how implementation should be conducted and scaled. WHO will also continue to explore opportunities to develop derivative products and evidence related to implementation of meaningful engagement through a scientific advisory group including communities living with NCDs, and mental health and neurological conditions, as well as experts from other health areas and sectors. Policy briefs will also be co-developed in collaboration with regional offices, which will support in translating WHO’s normative guidance around meaningful engagement into clear and impactful strategies and practices that meet the needs of regions and countries. In parallel, implementation projects will be established at the country level, including a needs assessment, starting in the WHO Eastern Mediterranean Region at the request and priority of the regional office.

### Conclusion

The upcoming fourth UN High-Level Meeting on NCDs in 2025 will stand as a global test of meaningful engagement and the transformation approach set out by the Framework. The process to develop the Framework unearthed and cemented strong interest and commitment to the principles of meaningful engagement and the process to co-create transformative health solutions with individuals with lived experience. People with lived experience have been leading the way to promote meaningful engagement, reorienting power to the people and communities most affected by these conditions. Whilst the Framework is a step forward by identifying clear principles, enablers, and recommendations, more commitment and investment is needed. This includes both WHO, governments, and other relevant stakeholders to further implement, test, and evaluate implementation of meaningful engagement across regions, further contextualizing and adapting the framework, and aligning with efforts to decolonize global health and achieve health for all.
